# Forced Diuresis with Matched Isotonic Intravenous Hydration Prevents Renal Contrast Media Accumulation

**DOI:** 10.3390/jcm11030885

**Published:** 2022-02-08

**Authors:** Yael Ben-Haim, Ehud Chorin, Aviram Hochstadt, Merav Ingbir, Yaron Arbel, Shafik Khoury, Amir Halkin, Ariel Finkelstein, Shmuel Banai, Maayan Konigstein

**Affiliations:** 1 Department of Cardiology, Tel Aviv Medical Center, Tel Aviv 6423906, Israel; yael@ben-haim.com (Y.B.-H.); udichorin5@gmail.com (E.C.); aviramho@gmail.com (A.H.); yarona@tlvmc.gov.il (Y.A.); kmail80@gmail.com (S.K.); amirh@tlvmc.gov.il (A.H.); afinkel@tlvmc.gov.il (A.F.); shmuelb@tlvmc.gov.il (S.B.); 2 Sackler School of Medicine, Tel Aviv University, Tel Aviv 6997801, Israel; merav2101@gmail.com; 3 Internal Medicine Department, Tel Aviv Medical Center, Tel Aviv 6423906, Israel

**Keywords:** contrast induced acute kidney injury, RenalGuard, renal failure, contrast media, forced diuresis

## Abstract

The accumulation of contrast media in the kidneys might lead to contrast-induced acute kidney injury. In this prospective, controlled observational study, we aimed to evaluate whether forced diuresis with matched isotonic intravenous hydration prevents the accumulation of contrast media in the kidneys of patients undergoing cardiac interventional procedures. We compared the intensity of contrast media accumulation as observed in nephrograms following these procedures, with and without peri-procedural controlled renal flushing. The study group consisted of 25 patients with impaired renal function treated with the RenalGuard system. The two control groups included 25 patients with normal kidney function and 8 patients with impaired renal function undergoing similar procedures with routine pre-procedural hydration, but without controlled renal flushing. Renal contrast media accumulation at the end of each procedure was scored by blinded cardiologists. The renal contrast accumulation score (CAS) in the study group was significantly lower, with a median score of 0 (IQR (0–0)) compared with 1.5 (IQR (1–2)) in the normal renal function control group and 1 (IQR (0.38–1.62)) in the impaired renal function control group (*p* < 0.001 and 0.003, respectively). In a multivariate analysis of CAS, RenalGuard treatment was independently associated with lower CAS compared to both control groups. In conclusion, RenalGuard use prevents renal contrast accumulation in patients with impaired renal function undergoing cardiac procedures with intra-arterial contrast media injection.

## 1. Introduction

Contrast-induced acute kidney injury (CI-AKI) is a known complication of coronary angiography and is associated with significantly unfavorable outcomes, including major cardiovascular events, the need for renal replacement therapy, prolonged hospitalization, and early death [1]. Data from the National Cardiovascular Data Registry (NCDR) CathPCI registry show that approximately 7% of patients undergoing PCI develop CI-AKI, mostly acute kidney injury (AKI) class 1, which is defined as an absolute rise in serum creatinine by ≥0.3 mg/dL or a 1.5–2-fold relative increase, according to the Acute Kidney Injury Network (AKIN) classification [2].

As there is no available effective therapy once acute kidney injury has occurred, the primary focus is to identify effective preventive therapies. The RenalGuard system (PLC Medical Systems, Milford, MA, USA) [3] causes controlled renal flushing by matching the IV infusion of an isotonic saline solution to furosemide-forced diuresis. Previous studies have shown that patients treated with the RenalGuard system showed lower incidence of CI-AKI [3,4,5]. Interestingly, several studies have demonstrated that the volume of contrast media used during the procedure does not correlate with the incidence of CI-AKI [5,6,7]. This finding may suggest that other factors, such as contrast media concentration once reaching the kidneys and the duration of kidney exposure to contrast may contribute to the development of AKI.

The purpose of this study was to provide a possible mechanism through which controlled renal flushing using forced diuresis with matching IV infusion of isotonic saline solution prevents CI-AKI in patients undergoing cardiac procedures involving intra-arterial contrast media injection. Furthermore, we wished to settle the discrepancy between the fact that the contrast media is nephrotoxic, yet the volume used has only a partial and limited effect on the incidence of CI-AKI. In the present study, we compared the radiographic intensity of contrast media accumulation in the kidneys following interventional cardiac procedures, with and without peri-procedural controlled renal flushing. We hypothesized that peri-procedural controlled renal flushing will prevent contrast media accumulation in the kidneys of patients who are at high risk of developing CI-AKI.

## 2. Materials and Methods

### 2.1. Study Design

This is a single center, observational, prospective, controlled study assessing the effect of peri-procedural controlled renal flushing on renal contrast media accumulation in patients at high risk of developing CI-AKI undergoing cardiac procedures involving intra-arterial contrast media injection. The study group consisted of consecutive patients referred for TAVR, coronary angiography, or angioplasty in our institution. All were considered at high risk of developing CI-AKI due to their impaired renal function (30 < eGFR < 60 mL/min/1.73 m^2^, or serum creatinine 1.5–2.5 mg/dL) and were pre-treated with the RenalGuard system, as per the standard of care at our center. The two control groups included the following: (1) Stable patients with normal renal function referred for the same cardiac interventional procedures with no RenalGuard treatment, and (2) patients with impaired renal function (30 < eGFR < 60 mL/min/1.73 m^2^, or serum creatinine 1.5–2.5 mg/dL) at high risk to develop CI-AKI, who did not receive RenalGuard treatment either due to difficulty in catheter placement, patient’s refusal to urinary catheter insertion, or operator’s decision. As RenalGuard use is the standard of care at our institution for patients with renal failure who undergo coronary angiography, PCI, or TAVI, we offered this treatment to all eligible patients, and those included in the second control group were only those who refused the treatment or were unable to receive it. 

Exclusion criteria were pregnancy, patients younger than 18 years, patients on chronic dialysis or with eGFR < 30 mL/min/1.73 m^2^ or creatinine > 2.5 mg/dL, patients who had received renal transplantation or nephrectomy, hemodynamically unstable patients, patients with acute STEMI, patients with severe symptomatic heart failure, and patients with LVEF < 30%. Excluded from the final analysis were patients who received less than 80mL of contrast media.

All patients provided written informed consent for participation in the study. The study was approved by the institutional ethics committee, in accordance with the ethical standards laid down in the 1964 Declaration of Helsinki and its later amendments. 

### 2.2. Study Protocol

Subjects in the study group were connected to the RenalGuard system as previously described in detail [4]. Briefly, following Foley catheter and vein line insertion, a pre-procedural IV bolus of 250 mL normal saline was administered over 30 min. After this priming, IV furosemide (0.25–0.5 mg/kg) was administered to achieve the recommended urine flow rate (UFR) of ≥300 mL/h. Once the target UFR was achieved, patients were transferred to the catheterization laboratory for the procedure. Controlled hydration by the system was continued during the procedure (procedural stage) and for an additional 4 h after the completion of the procedure (post-procedural stage), with UFR monitored and maintained throughout the procedure and during the following four hours, with additional furosemide doses as needed to maintain the recommended UFR (with increments of 0.25 mg/kg every thirty minutes). After the procedure, the IV saline infusion was maintained according to hemodynamic conditions to complete 12 h of infusion after contrast media administration. 

Patients in the control groups were treated with IV normal saline solution prior to the procedure according to their hemodynamic condition, and according to the operator’s pre-procedural medical instructions. After completion of the procedure, IV saline was continued for 12 h, unless otherwise instructed by the treating physician in accordance with the patient’s clinical status. 

The contrast media used in all patients, regardless of the type of procedure (angiography, PCI, or TAVR), were Iodixanol (Visipaque, GE Healthcare Ireland, Cork, Ireland; 290 mOsm/kg water). 

Renal radiographs of both kidneys (cine field: vertebra D11 to iliac crest bilaterally for 2 s, at 25 SD, <100 FD) were performed at the end of the procedure. An additional radiograph of the urinary drainage bag was performed at the end of the procedure for patients treated with RenalGuard system in order to document the presence of contrast media in the urine, showing that the contrast had passed through the kidneys. The total duration of the renal radiographs performed for the study was measured for each patient (study-related cine time) and compared to the total procedure cine time.

### 2.3. Renal Contrast Accumulation Analysis

**Figure 1:**
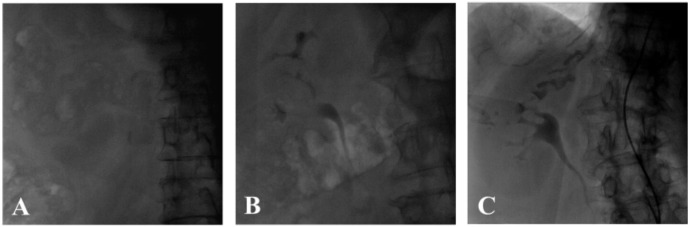
Kidney and collecting system contrast media accumulation score: (**A**) score 0: no renal or collecting system contrast accumulation; (**B**) score 1: mild contrast accumulation in the collecting system, without clear demarcation of the kidney; (**C**) score 2: intense contrast accumulation in the collecting system and the kidney.

Radiographs of the kidney and collecting system were viewed and scored by two independent, blinded cardiologists (S.K. and M.K.). Scoring of contrast media accumulation was determined on a scale of 0–2 as follows: score 0—no renal or collecting system contrast accumulation observed; score 1—mild contrast accumulation in the collecting system, without clear demarcation of the kidney; score 2—intense contrast accumulation in the collecting system and the kidney (Figure 1). In case of any disagreement between the two observers, a third blinded observer (Y.A.) scored the kidney contrast accumulation and the result used was the one given by two of the three observers.

### 2.4. Statistical Analysis

Between-group differences of continuous variables were assessed using a Wilcoxon test, while differences of categorical variables were assessed using a Fisher’s exact or a chi-square test, as appropriate. Inter-rater agreement between the two observers was assessed using a weighted kappa test using quadratic weights. In all cases where the two observers did not agree, a third observer was asked to rate the renal radiograph and the result used was the one given by the majority of observers. The final score per subject was the average of the scores received for each kidney. To diminish the effect of confounders, a multivariate analysis of the effect of renal guard treatment was performed using an ordinal regression model utilizing a logit link function with treatment, age, gender, presence of hypertension, diabetes, eGFR, contrast volume used, procedure type, and total procedure time as the independent variables, and average score as the dependent variable. The results are shown as mean (SD) or median (IQR), as appropriate. Differences were considered statistically significant when *p* < 0.05. All of the calculations were done using R-version 3.3.2 from R Foundation for Statistical Computing, Vienna, Austria.

## 3. Results

### 3.1. Study Population and Procedure Data

Final analysis was performed on a total of 58 patients (74.4 years, 77% male), with 25 in the study group, 25 in the normal renal function control group, and 8 in the impaired renal function control group. One patient was excluded from the study group due to low UFR while connected to the RenalGuard system. Eight patients, two from the normal renal function control group and six from the study group, received less than 80mL contrast media and were therefore also excluded from the study.

The study group consisted of a total of 25 patients who underwent angiography (32%), angioplasty (44%), or TAVR (24%). The baseline clinical characteristics are listed in Table 1. Patients in the study group presented a higher prevalence of hypertension and diabetes than the normal renal function control group (100% vs. 78% for hypertension and 60% vs. 21% for DM, *p* = 0.047 and 0.012, respectively). The mean baseline serum creatinine level was higher in the study group compared with the normal renal function control group (1.7 ± 0.5 mg/dL vs. 0.95 ± 0.27 mg/dL, respectively, *p* < 0.001). 

In contrast, the patients in the study group and in the impaired renal function control group were comparable in terms of baseline characteristics (mean age 76 ± 8.48 vs. 76 ± 10.73, respectively, *p* = 0.9). The prevalence of hypertension and diabetes were also similar in the two groups (100% hypertension in both groups, 60% DM in the study group, and 63% in the impaired renal function control group, *p* = 1.0). The mean baseline creatinine was also similar for the two groups (1.71 ± 0.5 mg/dL vs. 1.63 ± 0.13 in the impaired renal function control group, *p* = 0.65).

Procedure related data and RenalGuard parameters are listed in Table 2. The study group received a mean of 1279.5 ± 529.5 mL of IV fluids during the pre-procedural and procedural stages, and achieved a mean urine flow rate of 596 ± 310.4 mL/h. 

The volume of contrast media administrated was lower in the study group without reaching statistical significance (134.8 ± 44.04 mL vs. 152.52 ± 54.18 mL for study vs. control, respectively, *p* = 0.25, and 152.86 ± 69.49 mL for the impaired renal function control group, *p* = 0.41) mean procedure time was also similar for the three groups (43.00 ± 26.51 min, 35.94 ± 21.35 min, and 32.75 ± 37.05 min for the study, normal renal function control group, and impaired renal function control group, respectively, *p* = 0.46 and *p* = 0.52). The total additional study-related cine time was less than 1% of the total procedure cine among all of the patient groups. 

### 3.2. Contrast Accumulation Score

Two independent, blinded cardiologists scored the images according to the contrast accumulation score specified above. For the 58 patients included in the study, there were 115 renal radiographs at the end of the procedure, as one patient from the normal renal function control group was missing a radiograph of his left kidney. There were 81 agreements and 34 disagreements between the observers, with good inter-observer agreement and a weighted kappa of 0.80 (95% CI 0.75–0.853). The third observer agreed with observer 1 in 20 cases and with observer 2 in 14 cases.

**Figure 2:**
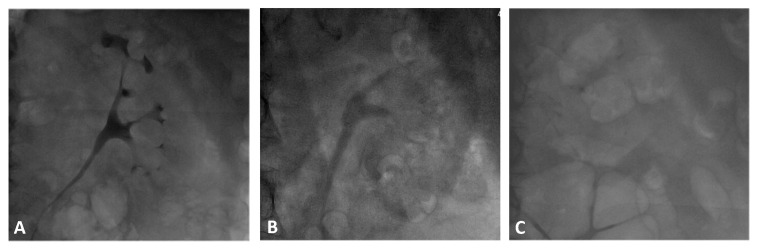
Comparison of renal contrast accumulation between patients in both groups. (**A**) 76-year-old male, creatinine 0.88 mg/dL, 175 mL contrast media; (**B**) 73-year-old male, creatinine 1.8 mg/dL, 194 mL contrast media; (**C**) 77-year-old male, creatinine 1.96 mg/dL, 164 mL contrast media, on RenalGuard.

**Figure 3:**
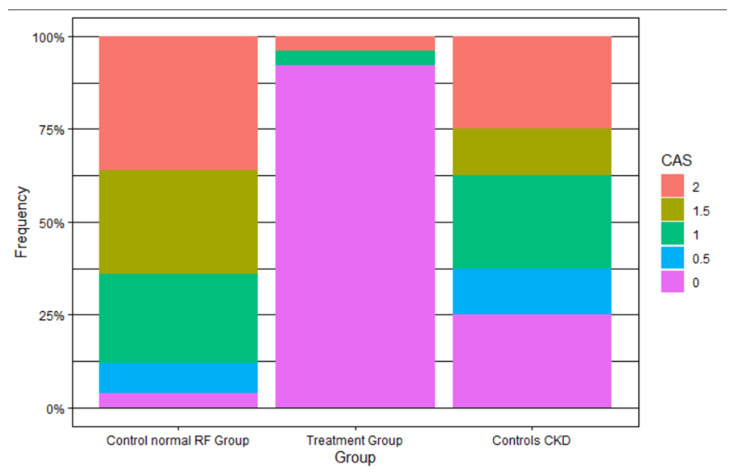
Contrast accumulation score (CAS) in study and control groups. CAS was significantly lower in the study group with a median score of 0 compared with 1.5 in the normal renal function control group and 1 in the impaired renal function control group, *p* < 0.001 and 0.003, respectively.

Examples of renal contrast accumulation in patients from the study and normal renal function control groups are presented in Figure 2. In the vast majority (92%) of patients treated with the RenalGuard system, the contrast accumulation score was 0 compared to only 4% in the normal renal function control group and 25% in the impaired renal function control group, *p* < 0.001 and 0.001, respectively, for the study vs. control groups. The contrast accumulation score (CAS) was significantly lower in the study group, with a median score of 0 (IQR (0–0)) compared with 1.5 (IQR (1–2)) in the normal renal function control group and 1 (IQR (0.38–1.62)) in the impaired renal function control group, *p* < 0.001 and 0.003, respectively (Figure 3). There was no significant difference in the CAS between the two control groups, *p* = 0.24. Contrast media were shown in the urinary drainage bag radiographs at the end of procedure in all RenalGuard treated patients. 

A multivariable analysis was performed as well. This analysis also showed a significant effect of RenalGuard use on CAS, with a significantly increased chance for lower CAS in the RenalGuard group compared with the two control groups (Table S1). 

### 3.3. Post Procedural Follow Up

Our study was not designed to detect differences in AKI in the control and study groups, and long term follow up is lacking. Altogether, 27 patients had follow-up data regarding renal function—11 in the normal renal function control group and 16 in the study group. The mean follow up time for the entire population was 1.8 ± 0.9 days, and was similar for both groups (*p* = 0.52). Change in GFR values from baseline to follow up was −0.6 ± 6.1 mL/min/1.73 m^2^ for the control group (*p* = 0.40) and 2.7 ± 7.8 mL/min/1.73 m^2^ for the treatment group (*p* = 0.32), with no significant difference between the groups (*p* = 0.15). The change in serum creatinine levels between baseline and follow up was −0.015 ± 0.11 mg/dL for the control group (*p* = 0.40) and −0.046 ± 0.37 mg/dL for the study group (*p* = 0.45), with no significant difference between the groups (*p* = 0.35).

## 4. Discussion

In this study, we showed that peri-procedural controlled renal flushing using forced diuresis with matched isotonic intravenous hydration, almost completely prevents contrast media accumulation in the kidneys of patients who underwent coronary angiography, angioplasty, or TAVR, and were considered at increased risk to develop CI-AKI. In 92% of the patients treated with controlled renal flushing, no contrast could be detected in the post procedural renal radiography. In contrast, among the control groups of patients with normal and impaired renal function, contrast media accumulation was clearly observed in renal radiography in 96% and 75% of the patients at the end of the procedure, respectively. Peri-procedural controlled renal flushing was significantly correlated with CAS, regardless of renal function, while there was no correlation between the volume of contrast media delivered and the degree of contrast accumulation, regardless of renal flushing.

Recently, McDonald et al. [8] demonstrated that persistent bilateral global nephrograms (visible kidneys in non-contrast CT) 24 h following contrast administration were detectable on non-contrast CT in patients who underwent contrast-enhanced CT or cardiac catheterization. This finding correlates with a marked increase in the likelihood of AKI (7-fold increase), dialysis (13-fold increase), or mortality (11-fold increase). Significant risk factors for nephrograms included factors leading to decreased renal perfusion, such as hypotension and shock. 

### 4.1. Pathophysiology of Contrast Induced Acute Kidney Injury

The pathophysiology of CI-AKI is not completely understood, but the current understanding highlights medullary ischemia as the pivotal mechanism [9]. Contrast media are cytotoxic and cause direct injury to the epithelial and tubular cells, which causes the release of vasoactive mediators, causing renal vasoconstriction and medullary ischemia. Furthermore, the high viscosity of contrast media may lead to capillary obstruction and decreased perfusion to the kidneys. Once contrast media are filtered and concentrated in the tubules, they may also obstruct them and cause further tubular damage [9,10].

Data regarding the role of contrast accumulation in the kidney, in the complex pathophysiology of CI-AKI, are lacking. It has been suggested in a rat model that contrast media administration increases the viscosity of the tubular fluid, thus leading to increased tubular pressure and impaired glomerular filtration. This prolongs the intrarenal retention time, and the longer exposure time might cause greater tubular damage, associated with higher renal toxicity [11].

### 4.2. Prophylactic Strategies for CI-AKI

Many preventive approaches for CI-AKI have been proposed and tested over the years. These include massive hydration, the use of low and iso-osmolar contrast media, and the administration of statins, sodium bicarbonate, and N-acetylcysteine [12]. The most widely used strategy is peri-procedural intravenous hydration with an isotonic saline solution [12,13]. The results of the AMACING trial, however, have challenged this routine approach by showing that a lack of prophylaxis was non-inferior for preventing CI-AKI compared with intravenous hydration, in a group of high-risk patients receiving contrast for radiological or cardiovascular procedures [14]. Moreover, hydration is often limited by potential complications of over-hydration, such as pulmonary congestion, especially in patients with chronic kidney disease, aortic stenosis, and heart failure. Over-hydration itself may increase the risk for AKI by increasing renal venous pressure and decreasing renal perfusion [15]. The RenalGuard system maintains an euvolemic state by inducing a high urine flow rate with matched IV hydration. The high urine flow rate may decrease the incidence of CI-AKI by reducing contrast media accumulation and by causing a rapid transit of contrast media through the kidneys. These two effects lead to lower overall kidney exposure to the contrast media, and potentially less medullary ischemia and less contrast media precipitation in the tubuli. Our study supports this suggested mechanism by showing a lower contrast accumulation score at the end of the procedure in patients treated with the RenalGuard system. 

### 4.3. Forced Diuresis with Matched Hydration Decreases the Incidence of CI-AKI Regardless of the Amount of Contrast Media Used

The REMEDIAL II trial [4] that included patients at very high risk for developing CI-AKI (eGFR < 30 mL/min/1.73 m^2^ and/or a Mehran risk score ≥ 11) demonstrated that RenalGuard therapy was superior to sodium bicarbonate and N-acetylcysteine administration in preventing CI-AKI. Moreover, the MYTHOS trial [3] demonstrated a significant reduction of CI-AKI rates, from 18% in the control group to 4.6% in the RenalGuard treated group. Similarly, we recently found that in patients considered at high risk to develop CI-AKI, undergoing coronary angiography, angioplasty, or TAVR, forced diuresis with matched controlled hydration resulted in decreased incidence of contrast induced AKI [5]. Conversely, in the REDUCE-AKI trial, the use of the RenalGuard during the TAVR procedure did not reduce the incidence of AKI and was associated with increased long-term mortality [16].

Previous studies showed no correlation between the contrast media volume and the development of CI-AKI [5,6,7]. In our study, there was no correlation between the amount of contrast media delivered and the contrast accumulation score at the end of the procedure. The lack of correlation between the two may indicate that other factors play a role in the development of CI-AKI, like contrast media concentration and the duration of exposure of the kidneys to the contrast media. The results of our study suggest that a shorter transit time of contrast media through the kidneys may have a protective effect and may reduce the risk of CI-AKI. As stated above, a lower median CAS in RenalGuard treated patients may indicate a shorter exposure of the kidneys to more dilute contrast media, which in turn may translate to a lower incidence of contrast induced AKI. 

### 4.4. Limitations

Our study has several limitations. First, we did not have data regarding the amount of IV fluids administered to the control group before or during the procedure. In addition, we did not systematically collect creatinine and electrolyte levels following the procedure, and therefore we could not provide data regarding the prevalence of CI-AKI among the three groups, nor data regarding the safety of forced diuresis, as our study was not designed to address these issues. Our second control group (of patients with impaired renal function undergoing the procedure without RenalGaurd system) included a small number of patients. Finally, we did not have data regarding long term sequela and complications of the procedure and/or the RenalGuard system. 

## 5. Conclusions

Peri-procedural controlled renal flushing using forced diuresis with matched intravenous hydration in patients undergoing interventional cardiac procedures involving intra-arterial contrast media administration is associated with decreased renal contrast media accumulation. Previous studies have shown that the use of forced diuresis with matched intravenous hydration is a safe and effective way to prevent CI-AKI in patients undergoing coronary angiography, angioplasty, or TAVR. The results of our study suggest that a shorter exposure time of the kidneys to more dilute contrast media is a possible mechanism of action.

## Figures and Tables

**Table 1:** Baseline patient characteristics.

	RenalGuard (*n* = 25)	Control Normal RF (*n* = 25)	Control Impaired RF (*n* = 8)	*p*-Value (RG vs. Control Normal RF)	*p*-Value (RG vs. Control Impaired RF)
Age (years ± SD)	76.20 ± 8.48	71.48 ± 13.97	75.75 ± 10.73	0.21	0.90
Female gender (%)	28	28	12.5	1	0.68
Creatinine (mg/dL ± SD)	1.71 ± 0.50	0.95 ± 0.28	1.63 ± 0.13	<0.001	0.66
eGFR (mL/min/1.73 m^2^± SD)	39.10 ± 13.91	76.04 ± 18.74	39.56 ± 6.61	<0.001	0.93
HTN (%)	100	78	100	0.047	NA
Hyperlipidemia (%)	76	79	87	1	0.85
DM (%)	60	21	63	0.012	1
TIA/CVA (%)	16	24	0	0.73	0.56
Tobacco use				0.47	0.72
Never (%)	53	31	50		
Past-smoker (%)	35	54	50		
Active smoker (%)	12	15	0		
AF (%)	20	30	13	0.62	1
HF (%)	36	13	50	0.13	0.77
IHD				0.06	0.21
No (%)	24	48	0.0		
s/p PCI (%)	44	44	75		
s/p CABG (%)	32	8	25		
Medications					
Beta Blockers (%)	67	50	71	0.38	1
CCB (%)	29	33	43	1	0.82
Statins (%)	75	71	71	1	1
ACE-I (%)	42	50	29	0.77	0.85
ARB (%)	25	13	29	0.46	1
PPI (%)	33	42	100	0.77	0.007
Aspirin (%)	71	67	86	1	0.76
Clopidogrel (%)	17	25	14	0.72	1
Ticagrelor (%)	0	0	14	NA	0.51
Prasugrel (%)	0	4	0	1	NA
Anticoagulation				0.13	
Warfarin (%)	0	8	0		
Rivaroxaban (%)	4	13	0		
Dabigatran (%)	0	4	0		
Apixaban (%)	13	0	14		

RF—renal function; eGFR—estimated glomerular filtration rate; HTN—hypertension; DM—diabetes mellitus; TIA—transient ischemic attack; CVA—cerebrovascular accident; AF—atrial fibrillation; HF—heart failure; IHD—ischemic heart disease; PCI—percutaneous coronary intervention; CABG—coronary artery bypass graft; CCB—calcium channel blocker; ACE-I—angiotensin converting enzyme inhibitor; ARB—angiotensin II receptor blocker; PPI—proton pump inhibitor.

**Table 2:** Procedure details.

	RenalGuard (*n* = 25)	Control Normal RF (*n* = 25)	Control Impaired RF (*n* = 8)	*p*-Value (RG vs. Control Normal RF)	*p*-Value (RG vs. Control Impaired RF)
Procedure				0.37	0.64
Diagnostic (%)	32	20	25		
PCI (%)	44	64	62.5		
TAVR (%)	24	16	12.5		
Total IV fluids (mL ± SD) *	1279.58 ± 529.52				
Total urine volume (mL ± SD) *	1067.32 ± 512.05				
Urine rate at end of procedure (mL/h ± SD) *	596.00 ± 310.44				
Total contrast volume (mL ± SD)	134.80 ± 44.04	152.52 ± 54.18	152.86 ± 69.49	0.25	0.41
Total procedure time (mins ± SD)	43.00 ± 26.51	35.94 ± 21.35	32.75 ± 37.05	0.46	0.52
Total procedure cine (s ± SD)	769.47 ± 597.50	689.24 ± 480.50	870.88 ± 527.41	0.71	0.69
Research cine (s ± SD)	7.13 ± 2.50	5.94 ± 1.89	3.75 ± 0.71	0.23	0.001

* Total IV fluids administered and total urine volume during the pre-procedural and procedural stages. RF—renal function; PCI—percutaneous coronary intervention; TAVR—transcatheter aortic valve replacement.

## Data Availability

The data presented in this study are available upon request from the corresponding author. The data are not publicly available due to privacy restrictions.

## Supplementary Materials

The following is available online at https://www.mdpi.com/article/10.3390/jcm11030885/s1, Supplementary file: Table S1: Multivariable analysis of mean contrast accumulation score as a function of various parameters.
