# Supplementary material for: Forced Diuresis with Matched Isotonic Intravenous Hydration Prevents Renal Contrast Media Accumulation
Source: J Clin Med. 2022 Feb 8;11(3):885. doi: 10.3390/jcm11030885 (PMC8837041; doi:10.3390/jcm11030885)
Supplement: Supplementary file 1 [file jcm-11-00885-s001.zip › jcm-1571860-supplementary.pdf]

Supplementary material

Supplementary Table S1

Multivariable analysis of mean contrast accumulation score as a function of various parameters

	OR [95% CI]	p-value
RenalGuard	1 (reference)	NA
Control (normal RF)	843.76 [15.86 to 278229.59]	0.005
Control (impaired RF)	126.99 [3.9 to 12782.82]	0.014
Procedure time (mins)	1.1 [1.04 to 1.2]	0.006
eGFR (ml/min/1.73m^2)	1.04 [0.97 to 1.11]	0.27
Procedure - PCI	2.82 [0.21 to 43.28]	0.44
Procedure - TAVR	2.05 [0.04 to 138.74]	0.72
Total contrast media (ml)	0.98 [0.94 to 1.01]	0.18
Female gender	0.99 [0.11 to 8.03]	1
Patient age (years)	0.93 [0.82 to 1.06]	0.28
Diabetes mellitus	0.69 [0.06 to 9.19]	0.77
Hypertension	0.6 [0.02 to 21.92]	0.77

RF – renal function, eGFR – estimated glomerular filtration rate, PCI - percutaneous coronary intervention, TAVR - transcatheter aortic valve replacement.

Multivariable analysis showed that RenalGuard treatment and procedure duration were the only factors independently associated with a lower CAS (OR 843.76, 95% CI 15.86 to 278229.59 p=0.005 for RenalGuard vs. normal renal function without RenalGuard, and OR 126.99, 95% CI 3.9 to 12782.82, p=0.014 for RenalGuard vs. impaired renal function without RenalGuard; OR 1.1, 95% CI 1.04 to 1.2, p=0.06 for procedure time). All variables were tested for interaction with treatment group by separate models with each specific interaction term. No interaction was found to be statistically significant ($p>0.05$), thus, all interaction terms were removed from the final model to enhance model predictability.
